# Unlocking the Ability of Generative Artificial Intelligence to Improve Health Communication and Health Education

**DOI:** 10.5888/pcd23.260490

**Published:** 2026-11-11

**Authors:** Kanhai S. Amin, David L. Skaggs, Rushabh H. Doshi, Pavan Khosla, Sanjiv Amin, Linda Mayes

**Affiliations:** 1 Yale School of Medicine, New Haven, Connecticut; 2 Yale Child Study Center, Yale School of Medicine, New Haven, Connecticut; 3 Department of Orthopedic Surgery, Cedars-Sinai Medical Center, Los Angeles, California; 4 Department of Internal Medicine, University of Southern California, Los Angeles, California; 5 Department of Pediatrics, Central Michigan University, Mount Pleasant, Michigan

## Abstract

**Introduction:**

While large language models (LLMs) can simplify information for patients, they are often ineffective at reaching specific reading levels. Here, we demonstrate a novel approach that may help improve readability and aid in the dissemination of health information to children and adolescents.

**Methods:**

We selected for analysis 200 common and diverse pediatric conditions and symptoms. We used ChatGPT to generate 36 explanations for each condition and symptom. For each explanation, we calculated reading grade levels (RGLs) and word counts. From the 36 outputs, we then selected single outputs for 5th-6th grade (level 1), 7th-8th grade (level 2), 9th-10th grade (level 3), and 11th-12th grade (level 4) by selecting outputs within 0.25 RGLs of the median of the grade range (ie, 5.25–5.75 for level 1). Three full professors then reviewed outputs related to their pediatric specialties for accuracy and completeness; they modified all incorrect information and added relevant missing information. We then tabulated RGLs, word counts, and the number of modifications for the edited outputs.

**Results:**

Prompt architectures varied in how well they initially resulted in a desired RGL, and few prompts resulted in requested RGLs at baseline. After output selection and accuracy edits, median RGLs were 5.8 (5.4–6.4) for level 1, 7.5 (7.4–7.7) for level 2, 9.5 (9.4–9.7) for level 3, and 11.5 (11.3–11.7) for level 4. Accuracy edits were minimal, with 93.5%, 95.5%, 95.5%, and 95.0% of outputs requiring no word additions or deletions for levels 1 through 4, respectively.

**Conclusion:**

Our approach offers health care providers a way to generate health information at a chosen reading grade level throughout the care continuum.

**Summary**

**What is already known on this topic?**

Large language models are increasingly used to communicate health information to patients but often fail to produce materials at appropriate reading grade levels.

**What is added by this report?**

We developed and evaluated a scalable approach for generating accurate explanations of pediatric conditions across 4 reading grade levels.

**What are the implications for public health practice?**

Our approach may help clinicians tailor educational and summary materials to children, people with limited health literacy, and other populations with special informational needs.

## Introduction

Health literacy is essential to ensuring safe, high-quality health care delivery, as emphasized by organizations including the Joint Commission, the American Academy of Pediatrics, the Centers for Disease Control and Prevention, and the US Department of Health and Human Services (1,2). Since the term “health literacy” was first introduced in 1974, the link between low health literacy and adverse health outcomes has become clear (3,4). For example, greater health literacy has been shown to improve medication adherence and reduce hospital admissions among adults (5–7).

Emerging research suggests that improved health literacy may also benefit children. Up to 85% of children and adolescents demonstrate low health literacy (1,8,9). Better health literacy in this population has been linked to smoother transitions to adult care, better self-management, and improved communication with health care providers, especially for chronic conditions such as congenital heart disease, spina bifida, cancer, and rheumatic diseases (10–14). Addressing health literacy among children and adolescents with chronic conditions, the prevalence of which is estimated as 8% to 25% among those aged 11 to 15 years (approximately grades 5–10), is particularly important, because at this age, many begin taking on self-care responsibilities (15,16).

Various strategies have been proposed to enhance health literacy, including patient portals, visual aids, and “show-me” or “teach-back” methods (17). Writing health information at lower reading grade levels (RGLs) — or levels tailored to specific patient needs — may also improve patient comprehension (18,19). However, many of these health interventions are time-consuming and expensive, particularly given that effective health literacy interventions often need to be tailored to the individual needs of patients (20). 

Recently, large language models (LLMs) and generative artificial intelligence (AI) technologies have gained attention as a way to improve health literacy by assisting with health communication and education (21–23). Despite suggestions that LLMs can tailor explanations to individual reading levels, research indicates limitations in this capability (21–23). Namely, LLMs cannot generate information at requested RGLs, and information accuracy requires further analysis before dissemination to patients (21–23). The objective of this study was to demonstrate a novel approach for generating medical information at specific RGLs that pediatricians, parents, counselors, and others can use to help explain information to children and adolescents. Although we tailored our approach for children and adolescents, similar methods can be used across the care continuum to better and readily explain information written at specific reading grade levels.

## Methods

We selected 200 common yet diverse pediatric conditions and symptoms across a range of medical specialties, including cardiology and vascular diseases; dental and oral health; dermatologic and craniofacial conditions, as well as plastic surgery; endocrinology; otorhinolaryngology; gastroenterology and hepatology; general pediatrics; genetics; hematology and oncology; immunology, allergy, and infectious diseases; neurology and psychiatry; obstetrics and gynecology; orthopedics and musculoskeletal conditions; pulmonology; and urology and nephrology (Appendix). We chose these conditions and symptoms while balancing prevalence, severity, and inclusion of difficult-to-explain conditions, drawing insights from the conditions and symptoms listed on the websites of Seattle Children’s Hospital and Johns Hopkins Children’s Center (24,25).

We used OpenAI’s ChatGPT application programming interface. We wrote and used 36 prompts to generate 36 outputs for each condition using zero-shot prompting (ie, providing no examples or finetuning). We used 3 general prompt architectures based on previous insights and OpenAI’s recommendations (22,23,26–28): Prompt A: “I am a []-grader and I read at the []-grade reading level. I need help understanding []”; Prompt B: “I am a physician and I need help explaining [] to a []-grader. Please provide output at the []-grade reading level”; and Prompt C: “You are a health literacy tool. Explain [] to a []-grader. Please provide output at the []-grade reading level.” We added “Please provide output at the [] reading level by using basic words and keeping sentences short” to Prompt B and Prompt C for the first- to fifth-grade levels given the external “I am a physician” and “You are a health literacy tool” nature of the prompt and previous findings (23).

For each of these 36 outputs per condition, we calculated RGLs and word counts. The RGL was calculated by averaging the Automated Readability Index, the Coleman–Liau Index, the Gunning Fog Index, and the Flesch–Kincaid Grade Level following previously established methods (22,23,26). These readability scales calculate RGLs using a combination of sentence length, word length, and syllables per word. 

For each condition, we selected 1 output for each of the following complexity levels: level 1, fifth to sixth grade; level 2, seventh to eighth grade; level 3, ninth to 10th grade; and level 4, 11th to 12th grade. Outputs were chosen if they fell within 0.25 RGLs of the median for each complexity level (eg, 5.25–5.75 for level 1). If multiple outputs met the criteria, we selected the output with the highest word count. If no output matched, we selected the output closest to the target RGL. After selection, we removed all extraneous information such as, “Sure I can help with that!”, “I hope that helps!”, and “Sure, I can explain at the []-grade level.” 

After removal of extraneous information, 3 full professors (D.L.S., S.A., L.M.) then reviewed outputs related to their pediatric specialties, correcting inaccuracies and adding missing information with recognition that outputs would be made available online at www.pediatricliteracy.org (28). Each of the 800 outputs was reviewed by at least 1 reviewer. We tabulated the number of words deleted and added to each output; we counted additions and deletions counted separately (eg, a single output could have 15 words added and 8 words deleted). After review, we recalculated RGLs and word counts.

In accordance with the Common Rule, this study did not require institutional review board approval because it did not involve human participants. We used Python version 3.11 (Python Software Foundation) to generate output, readability scores, and word counts, and to select outputs. We used R version 2023.12.1 (R Foundation for Statistical Computing) to perform Wilcoxon signed-rank tests; significance was set at an α of .05. We also used R to create graphs. All code is accessible online with instructions (29).

## Results

Prompt architectures varied in their ability to reach a specified RGL, and few outputs reached the RGL requested in the prompt (Table). The lowest median RGL of any prompt, 6.6, came from prompt 25, above the desired fifth- to sixth-grade target for complexity level 1. However, by generating 36 outputs for each condition, target RGLs, including for level 1, were achieved in most cases. The median (IQR) RGLs were 5.8 (5.4–6.4) for complexity level 1, 7.5 (7.4–7.7) for complexity level 2, 9.5 (9.4–9.7) for complexity level 3, and 11.5 (11.3–11.7) for complexity level 4, with significant differences between complexity levels (Figure 1). Word counts for selected outputs were 97.0 (75.0–166.3) for complexity level 1, 258.0 (177.0–337.3) for complexity level 2, 345.0 (286.8–395.5) for complexity level 3, and 357.0 (318.8–401.0) for complexity level 4, with significant differences between complexity levels (Figure 2).

**Table:** Output Reading Grade Level and Word Count of Prompts Submitted to ChatGPT to Generate Medical Information for Children and Adolescents and Number of Instances the Prompt’s Output was Chosen

Prompt no.	Prompt	Resulting RGL, median (IQR)^a^	Resulting word count (IQR)	Complexity^b^			
				Level 1 (5th-6th grade)	Level 2 (7th-8th grade)	Level 3 (9th-10th grade)	Level 4 (11th-12th grade)
1	I am a first grader and I read at the first-grade reading level. I need help understanding [].	10.5 (9.3–11.6)	324.0 (298.0–357.3)	0	1	8	5
2	I am a second grader and I read at the second-grade reading level. I need help understanding [].	10.5 (9.4–11.4)	329.5 (299.0–355.5)	0	0	13	6
3	I am a third grader and I read at the third-grade reading level. I need help understanding [].	10.9 (9.9–12.1)	340.0 (311.0–370.5)	0	2	8	4
4	I am a fourth grader and I read at the fourth-grade reading level. I need help understanding [].	11.3 (10.3–12.2)	348.0 (319.5–377.0)	0	0	5	17
5	I am a fifth grader and I read at the fifth-grade reading level. I need help understanding [].	11.5 (10.5–12.3)	348.0 (321.0–376.5)	0	0	3	9
6	I am a sixth grader and I read at the sixth-grade reading level. I need help understanding [].	12.0 (11.1–12.8)	355.0 (325.0–384.3)	0	0	2	10
7	I am a seventh grader and I read at the seventh-grade reading level. I need help understanding [].	12.1 (11.1–13.3)	358.5 (330.8–389.0)	0	0	1	16
8	I am an eighth grader and I read at the eighth-grade reading level. I need help understanding [].	12.4 (11.3–13.2)	355.0 (321.8–388.0)	0	0	1	13
9	I am a ninth grader and I read at the ninth-grade reading level. I need help understanding [].	12.6 (11.6–13.7)	357.0 (330.0–389.0)	0	0	1	15
10	I am a tenth grader and I read at the tenth-grade reading level. I need help understanding [].	12.7 (11.7–13.6)	366.0 (338.0–403.3)	0	0	1	17
11	I am an eleventh grader and I read at the eleventh-grade reading level. I need help understanding [].	12.8 (11.9–13.9)	373.0 (346.0–406.3)	0	0	1	11
12	I am a twelfth grader and I read at the twelfth-grade reading level. I need help understanding [].	12.8 (12.1–13.9)	369.5 (344.0–402.3)	0	0	1	6
13	I am a physician and I need help explaining [] to a first grader. Please provide output at the first-grade reading level by using basic words and keeping sentences short.	7.1 (6.0–7.8)	102.0 (78.0–170.3)	23	7	1	0
14	I am a physician and I need help explaining [] to a second grader. Please provide output at the second-grade reading level by using basic words and keeping sentences short.	7.0 (6.1–7.9)	121.0 (85.0–190.0)	26	8	0	0
15	I am a physician and I need help explaining [] to a third grader. Please provide output at the third-grade reading level by using basic words and keeping sentences short.	7.3 (6.5–8.1)	124.5 (87.0–200.5)	17	7	0	0
16	I am a physician and I need help explaining [] to a fourth grader. Please provide output at the fourth-grade reading level by using basic words and keeping sentences short.	7.6 (6.8–8.4)	180.0 (106.5–220.0)	12	10	1	0
17	I am a physician and I need help explaining [] to a fifth grader. Please provide output at the fifth-grade reading level by using basic words and keeping sentences short.	7.7 (6.8–8.4)	185.5 (111.8–230.3)	10	9	1	0
18	I am a physician and I need help explaining [] to a sixth grader. Please provide output at the sixth-grade reading level.	8.4 (7.6–9.2)	288.5 (256.0–324.0)	0	12	9	1
19	I am a physician and I need help explaining [] to a seventh grader. Please provide output at the seventh-grade reading level.	8.6 (7.8–9.4)	296.0 (267.0–333.3)	0	14	10	0
20	I am a physician and I need help explaining [] to an eighth grader. Please provide output at the eighth-grade reading level.	9.1 (8.3–9.8)	289.0 (259.8–327.0)	1	6	16	1
21	I am a physician and I need help explaining [] to a ninth grader. Please provide output at the ninth-grade reading level.	9.3 (8.5–10.2)	310.0 (275.0–342.5)	0	12	14	3
22	I am a physician and I need help explaining [] to a tenth grader. Please provide output at the tenth-grade reading level.	9.7 (8.8–10.6)	299.5 (265.8–343.3)	0	2	10	6
23	I am a physician and I need help explaining [] to an eleventh grader. Please provide output at the eleventh-grade reading level.	10.2 (9.2–11.0)	326.0 (290.8–366.0)	0	0	11	12
24	I am a physician and I need help explaining [] to a twelfth grader. Please provide output at the twelfth-grade reading level.	11.0 (10.2–12.0)	344.0 (300.8–371.0)	0	1	5	11
25	You are a health literacy tool. Explain [] to a first grader. Please provide output at the first-grade reading level by using basic words and keeping sentences short.	6.6 (5.4–7.5)	79.0 (66.0–101.3)	43	12	0	0
26	You are a health literacy tool. Explain [] to a second grader. Please provide output at the second-grade reading level by using basic words and keeping sentences short.	7.0 (6.0–7.9)	89.0 (71.0–131.0)	22	7	0	0
27	You are a health literacy tool. Explain [] to a third grader. Please provide output at the third-grade reading level by using basic words and keeping sentences short.	7.2 (6.2–8.0)	98.0 (73.0–170.0)	21	10	1	0
28	You are a health literacy tool. Explain [] to a fourth grader. Please provide output at the fourth-grade reading level by using basic words and keeping sentences short.	7.4 (6.6–8.4)	105.5 (85.0–196.3)	9	6	1	0
29	You are a health literacy tool. Explain [] to a fifth grader. Please provide output at the fifth-grade reading level by using basic words and keeping sentences short.	7.4 (6.6–8.2)	113.5 (83.0–187.8)	12	12	2	1
30	You are a health literacy tool. Explain [] to a sixth grader. Please provide output at the sixth-grade reading level.	8.2 (7.3–9.1)	305.5 (249.0–405.3)	4	21	9	0
31	You are a health literacy tool. Explain [] to a seventh grader. Please provide output at the seventh-grade reading level.	8.3 (7.6–9.0)	305.5 (247.8–396.8)	0	16	5	0
32	You are a health literacy tool. Explain [] to an eighth grader. Please provide output at the eighth-grade reading level.	8.8 (7.9–9.6)	280.0 (232.0–332.0)	0	10	6	2
33	You are a health literacy tool. Explain [] to a ninth grader. Please provide output at the ninth-grade reading level.	9.3 (8.3–10.1)	314.5 (252.0–431.0)	0	9	15	6
34	You are a health literacy tool. Explain [] to a tenth grader. Please provide output at the tenth-grade reading level.	9.6 (8.6–10.7)	324.0 (257.0–431.3)	0	5	20	3
35	You are a health literacy tool. Explain [] to an eleventh grader. Please provide output at the eleventh-grade reading level.	10.1 (9.2–11.2)	344.5 (279.5–460.5)	0	0	11	8
36	You are a health literacy tool. Explain [] to a twelfth grader. Please provide output at the twelfth-grade reading level.	11.1 (10.0–12.1)	315.0 (266.5–414.5)	0	1	7	17

Abbreviation: RGL, reading grade level.

a Calculated by averaging the Automated Readability Index, the Coleman-Liau Index, the Gunning Fog Index, and the Flesch–Kincaid Grade Level following previously established methods (22,23,26).

b For each condition, we selected 1 output for each of the following complexity levels: level 1, fifth to sixth grade; level 2, seventh to eighth grade; level 3, ninth to 10th grade; and level 4, 11th to 12th grade. Table denotes number of times the prompt’s output was used when selecting 1 output per complexity level and condition/symptom.

**Figure 1:**
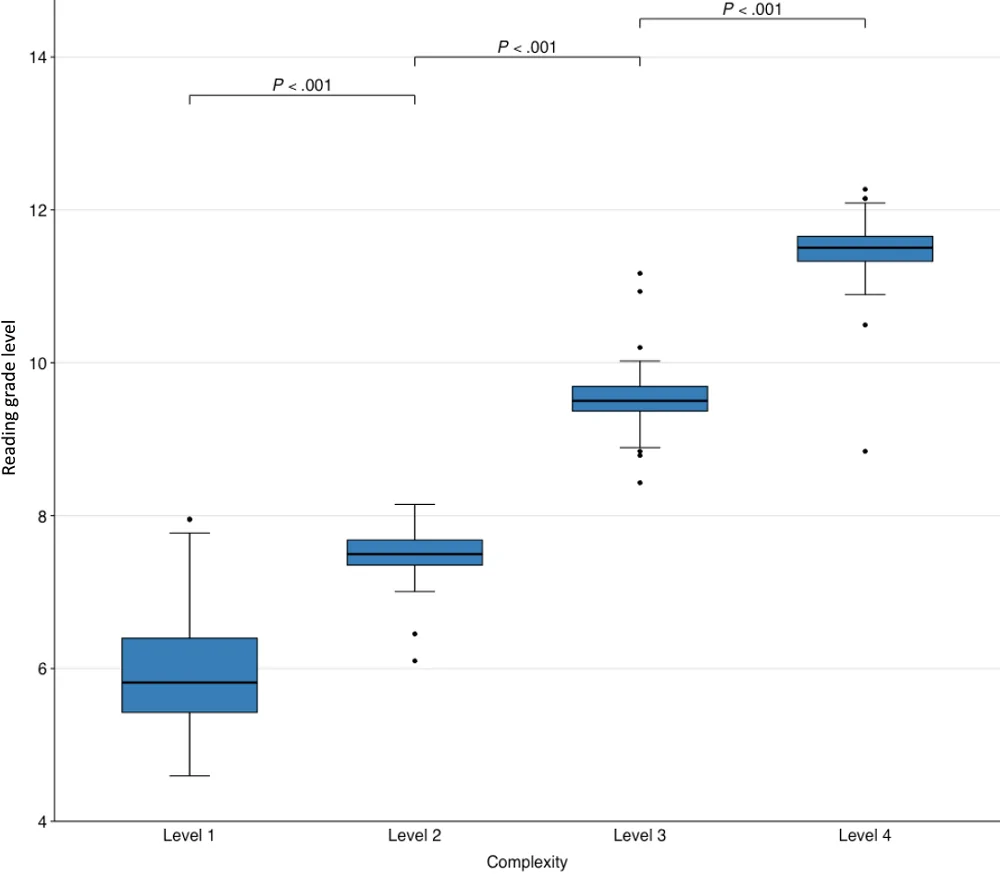
Reading grade levels for each complexity level. Boxes indicate the 25th percentile, median, and 75th percentile; dots indicate outliers; whiskers indicate 1.5 IQR.

**Figure 2:**
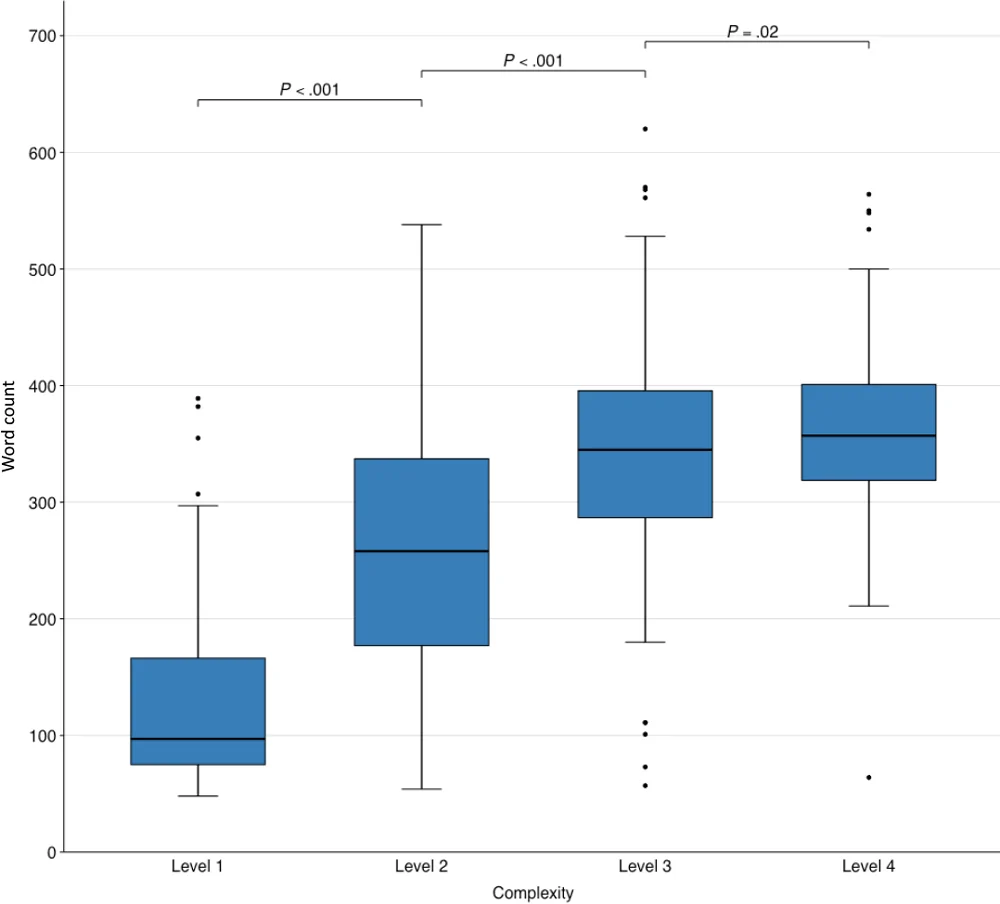
Word counts of optimized outputs for each complexity level. Boxes indicate the 25th percentile, median, and 75th percentile; dots indicate outliers; whiskers indicate 1.5 IQR.

Accuracy edits for LLM outputs were minimal, with 93.5%, 95.5%, 95.5%, and 95.0% of outputs for complexity levels 1 through 4, respectively, requiring no word additions or deletions (Figure 3). For outputs that required edits, median (IQR) word additions were 6 (2.8–11.0) for complexity level 1, 4 (1.5–5.5) for complexity level 2, 5 (1.0–9.0) for complexity level 3, and 3 (2.0–6.0) for complexity level 4. Word deletions were 8 (5.0–13.5) for complexity level 1, 10 (5.3–11.8) for complexity level 2, 4.5 (1.8–22.3) for complexity level 3, and 5 (12.5–20.8) for complexity level 4. Many changes were minor. For example, “Remember, our body is amazing and strong, and with the help of doctors, we can overcome any challenges we face, just like superheroes!” was revised to “Remember, our body is amazing and strong, and with the help of doctors, we can overcome these challenges!”

**Figure 3:**
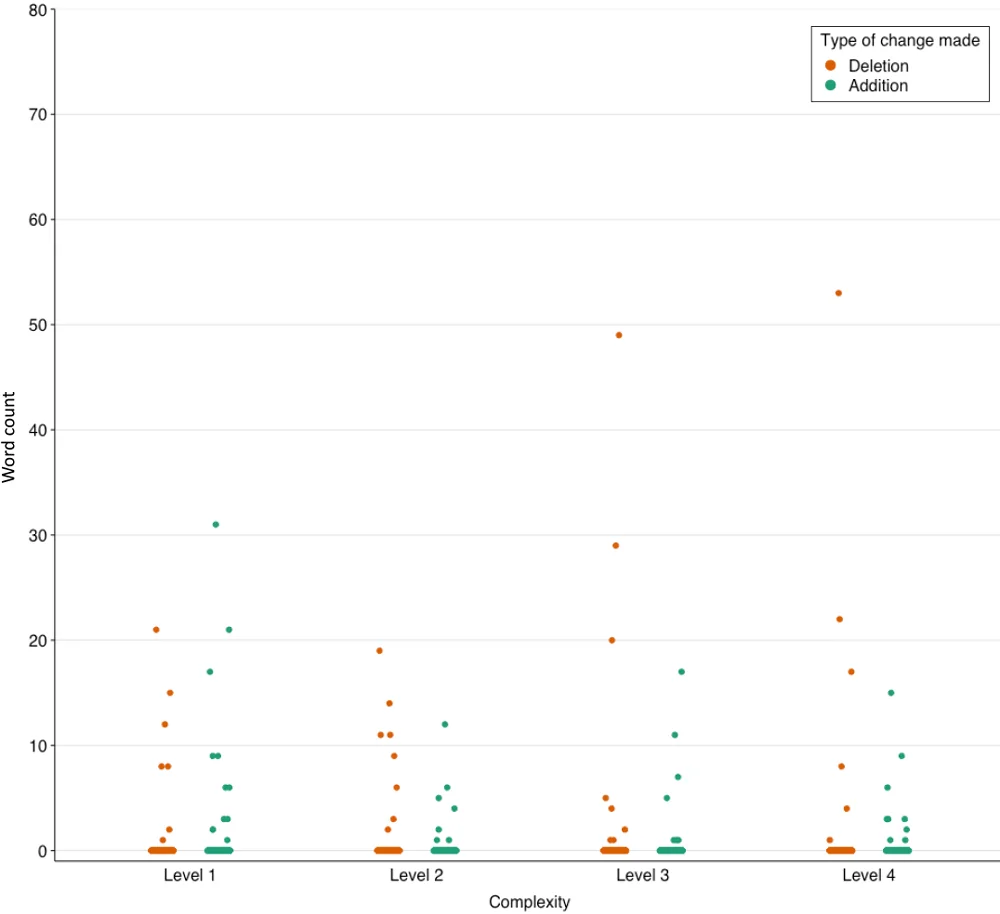
Jitter plot of the number of modifications made during expert review of medical information generated by prompts submitted to ChatGPT. Dots indicate the number of words reviewers deleted from and added to selected outputs. The short, thick lines at zero indicate the outputs that required no edits.

## Discussion

Consistent with prior findings, we found that the LLM with zero-shot prompting (ie, providing no examples or finetuning) struggled to produce outputs at the requested RGLs, especially for content below the seventh-grade level (22,23,26). This shortcoming likely stemmed from the LLM’s training data, which may have been biased toward higher RGLs. Other models, such as Google Gemini and Microsoft Bing, often produce output at the 10th- and 11th-grade levels at baseline, respectively, while ChatGPT frequently produces outputs at the college level (22,23). Despite this, we selected ChatGPT for its superior ability to vary output RGLs (22,23).

We anticipated that our model would have difficulty generating content at the requested RGLs, particularly at lower levels. However, by leveraging both the natural variation in LLM outputs and multiple prompt architectures, we were able to extract appropriate content for each reading level. Interestingly, framing the prompt with the persona of “you are a health literacy tool” and explicitly stating “by using basic words and keeping sentences short” improved the model’s ability to generate outputs down to the fifth-to-sixth–grade level (30). However, due to difficulties in generating lower reading-level output, complexity level 1 still had the largest deviation away from the target RGL of 5.5 and had an IQR of 5.4 to 6.4. For all other complexity levels, the IQRs were 0.2 RGLs away from the target complexity level, aligning with our output selection criteria (within 0.25 RGLs from the median). In future studies and applications, generating more than 36 outputs via an application programming interface or further prompt engineering could enhance the extraction of content at lower RGLs. Although zero-shot prompting is commonplace for LLMs, our findings suggest that generating multiple outputs followed by automated selection is the most likely to reach desired RGLs.

Because target reading levels for children are poorly understood (31), we used 4 complexity levels spanning a wide range, fifth grade to 12th grade. For adults, guidelines recommend that all adult-facing health information be written below the eighth-grade level, but these guidelines and readability scales often fail to consider prior knowledge, motivation, and context (22). Although RGLs are objective, they have limitations. Hence, we grouped RGLs (eg, fifth and sixth grades) into a single complexity level and generated outputs at 4 complexity levels, allowing users to choose output aligned with their needs.

Instead of using a Likert scale or other verification scales, we assessed accuracy by having expert reviewers ensure that the outputs had no incorrect or critical missing information before publication online (29). Although our process for assessing accuracy reflects some subjectivity, we believe that entrusting experts with ensuring accuracy and completeness is adequate and more relatable to the real world than other methods. For AI applications in health care, the process of engaging experts serves as the “human-in-the-loop” (32,33).

We found that our models accurately generated information, a finding that differs from those of several studies that examined requests for diagnostic decisions (21,34,35). For the limited outputs that required changes, the changes were minor. Our findings on accuracy may differ from findings on diagnostic accuracy because LLMs are generative AI technologies that predict the next word, which may increase their ability to provide useful definitions, explanations, or simplifications of medical information — particularly for terms that are likely to be in their training data. 

Regardless of prompt, the expert reviewers indicated that most outputs were accurate. This finding is reassuring because children and adolescents increasingly turn to the internet for medical advice (36). Three-quarters of adolescents report using the internet as their most recent source of medical information (37,38). However, for clinical use, experts verifying LLM outputs before sharing them with patients is essential. The ideal workflow for generating health information for children and adolescents would include pediatricians, school nurses, or other health care professionals, as well as verification of accuracy and completeness of outputs. Although we did not measure verification or review time, the minimal changes required in our study suggest that reviewing outputs is not time-intensive, especially given the typical reading speed of college-educated adults (200–400 words per minute) (39).

Although our study focused on pediatric health applications, the potential of our approach of readily generating multiple outputs and computationally selecting the desired complexity level extends to many segments of the health care continuum and to other fields. From generating patient-friendly imaging reports, discharge summaries, medication guidelines, surgical guidelines, and more, this approach may help realize the health communication goals of patient-centered medicine (21,26,28). These goals are particularly relevant in the era of the 21st Century Cures Act (40) which mandates immediate release of many types of health data (eg, imaging reports) to patients even though the jargon and complex technological terms contained in these data releases often cause patient confusion and anxiety (21,26,41). For example, if LLMs were used to simplify radiology reports for patients, they could generate reports tailored to the reading level of individual patients. However, the attitude of health care providers toward AI-generated simplifications requires further assessment (42).

Ultimately, we demonstrated that generating multiple outputs and computationally selecting ideal outputs corrected by experts in a field can improve the readability of health information for patients. Through this approach, we can empower patients with health information at the appropriate reading level, improving both patient engagement and health outcomes.

## Appendix Categories for 200 Conditions and Symptoms Commonly Found in Pediatric Populations in the US^a^

**Table:**

Category	Count
Cardiology and vascular diseases	22
Dental and oral health	3
Dermatologic conditions, craniofacial disorders, and plastic surgery	23
Endocrine Disorders	4
Otorhinolaryngological	4
Gastrointestinal and liver disorders	29
General pediatrics	2
Genetic	7
Hematology and oncology	17
Immunology, allergic disorders, and infectious diseases	24
Neurological and psychiatric disorders	26
Obstetrics and gynecology	3
Orthopedic and musculoskeletal disorders	30
Respiratory disorders	5
Urology and nephrology	1
**Total**	**200**

^a^ Conditions and symptoms were selected by balancing prevalence, severity, and inclusion of difficult-to-explain conditions and by referring to the websites of Seattle Children’s Hospital and Johns Hopkins Children’s Center (24,25).
